# Sperm DNA integrity does play a crucial role for embryo development after ICSI, notably when good-quality oocytes from young donors are used

**DOI:** 10.1186/s40659-022-00409-y

**Published:** 2022-12-26

**Authors:** Jordi Ribas-Maynou, Sergi Novo, Marc Torres, Albert Salas-Huetos, Sergi Rovira, Marta Antich, Marc Yeste

**Affiliations:** 1 Unit of Cell Biology, Department of Biology, Faculty of Sciences, University of Girona, C/ Maria Aurèlia Capmany 69, ES-17003 Girona, Spain; 2 Biotechnology of Animal and Human Reproduction (TechnoSperm), Institute of Food and Agricultural Technology, University of Girona, Girona, Spain; 3 Fertilab – Institut Catala de Fertilitat SL, Barcelona, Spain; 4 Fertibank, Barcelona, Spain; 5 Department of Nutrition, Harvard T.H. Chan School of Public Health, Boston, MA USA; 6 Catalan Institution for Research and Advanced Studies (ICREA), Barcelona, Spain

**Keywords:** Sperm, Male-factor, Infertility, Double donation, ICSI, Oocyte donors

## Abstract

Based on the inconsistent literature published thus far involving infertile patients, whether intracytoplasmic sperm injection (ICSI) allows overcoming total fertilization failure due to sperm DNA fragmentation is still unclear. Related to this, female factors, which may have a significant impact on assisted reproduction outcomes, can mask male infertility. In this scenario, evaluating ICSI outcomes following cycles using healthy donor gametes could shed light on this realm, as it would avoid the influence of (un)known confounding factors present in infertile individuals. The present work, therefore, aimed to address whether single- and double-stranded sperm DNA fragmentation leads to impaired ICSI outcomes in double gamete donation cycles. The study also compared these double-gamete donation cycles to cycles in which only sperm were donated and oocytes were obtained from infertile patients. Two cohorts were included: (a) the Donor-Donor (DD) cohort, which included 27 semen donor samples used in 49 ICSI cycles with young healthy oocyte donors; and (b) the Donor-Infertile (DI) cohort, which involved 34 semen donor samples used in 57 ICSI cycles with oocytes from patients. Single- and double-stranded sperm DNA breaks were determined with alkaline and neutral Comet assays, respectively; ICSI was conducted following standard protocols and embryos were monitored through time-lapse microscopy. In the DD cohort, the percentage of sperm with high overall DNA damage correlated with fertilization rates (Rs = − 0.666; *P* < 0.001) and with the percentage of blastocysts per injected oocyte (Rs = − 0.414; *P* = 0.040). In addition, sperm DNA damage delayed the first embryo division (Rs = 0.421; *P* = 0.036), and development from the 8-cell to the morula stage (Rs = 0.424; *P* = 0.034). In contrast, double-stranded DNA breaks had no effect in this cohort. As far as the DI cohort is concerned, while overall sperm DNA damage was not found to be correlated to fertilization or blastocyst rates, pronuclei formation following ICSI was delayed when the incidence of double-stranded DNA breaks was high (Rs = 0.485; *P* = 0.005). In conclusion, this study, which is the first involving double donation cycles (i.e., a donor-donor cohort), supports that sperm DNA damage has a detrimental impact on fertilization rates after ICSI, and delays embryo development. Moreover, the use of oocytes from infertile individuals is suggested to hide the male-factor effect.

**Supplementary Information:**

The online version contains supplementary material available at 10.1186/s40659-022-00409-y.

## Introduction

Delivering an intact paternal genome to the oocyte is one of the most important steps to ensure embryo development. In order to protect DNA from genotoxic damage, sperm chromatin is highly condensed during spermatogenesis, thanks to the replacement of most histones by protamines [1]. Although this high degree of condensation is useful to prevent enzymatic activity in most regions of sperm DNA, thus blocking the effects of nucleases [2], sperm cells may suffer genetic damage due to an intrinsic or external increase of reactive oxygen species, which causes oxidative stress at testicular and/or post-testicular stages [3]. Because gene transcription is completely interrupted in mature sperm cells, DNA alterations, such as oxidized or deaminated bases, adducts, crosslinks and single-strand or double-strand breaks, cannot be repaired. In this scenario, the capacity of oocytes to overhaul paternal DNA insults after fertilization is crucial to supply the future organism with a complete, uninterrupted and non-mutated genome [4–6].

In somatic cells, the presence of non-repaired DNA breaks in the genome is deleterious and induces apoptosis [7]. Regarding germ cells, mounting evidence in humans and animal models points out that DNA fragmentation in sperm is related to male-factor infertility, reducing the occurrence of natural pregnancy and increasing the number of attempts needed to conceive [8–10]. Yet, whether sperm DNA damage detrimentally affects embryo development following assisted reproductive treatments (ART) has been a topic of much debate among scientists and clinicians in the last decades. In effect, while some studies reported that sperm DNA fragmentation negatively affects embryo development in humans [11–16], others found no influence [17–21]. This controversy also underpinned the opposite recommendations given by the different human reproduction societies regarding the management of patients with high sperm DNA damage [22–24]. Hence, an unequivocal answer to this question is vital to establish whether this variable has a prognostic value for infertile couples; if so, this could help determine the most suitable ART treatment in each case.

On the way towards addressing this controversial matter, potential factors biasing data have been identified [22, 25]. First, female factors have to be taken into consideration when investigating male infertility. This is especially pertinent in studies including infertile couples, where impaired oocyte quality can either be competent to repair sperm DNA [26, 27], or bring additional alterations in embryo development [28]. Indeed, because oocytes are arrested at meiotic prophase I since fetal stages, they are more likely to accumulate alterations and exhibit reduced fertilizing ability in women with advanced age [29–32]. Second, alterations in sperm count, motility and morphology may result from unknown physiological or molecular alterations that ultimately affect embryo development [33–36]. Third, the assessment of sperm DNA integrity is not standardized among laboratories, and methods with superior sensitivity rather than the indirect ones may be needed to estimate DNA damage in sperm cells with highly condensed chromatin [37–40]. Also, whether DNA breaks are single or double should be considered as an important factor related to clinical outcomes [41–43]. Finally, methodological procedures such as the selection of a single sperm cell in ICSI versus IVF [44–47], or the uninterrupted embryo culture monitored under time-lapse versus sequential culture [48, 49] may cause a bias with respect to the whole sample measurement.

Studies conducted in animal models unequivocally show that induced sperm DNA fragmentation adversely affects IVF and ICSI outcomes [50]. In human infertile couples, the latest and largest meta-analysis including more than 12,000 ART cycles revealed that while sperm DNA fragmentation has a negative impact on assisted reproductive outcomes after conventional IVF, it does not have the same effect in ICSI; substantial heterogeneity and the existence of a publication bias could explain these non-conclusive results [25]. Conducting further research to shed light on whether sperm DNA damage alters ICSI outcomes is, therefore, much warranted.

The present work aimed to evaluate if DNA damage exerts a negative impact on assisted reproductive outcomes after ICSI, when donor sperm and oocytes from healthy young donors are involved. In addition, whether the impact of sperm DNA damage on embryo development changed when oocytes from infertile women were microinjected was also determined.

## Results

### Semen donor parameters and descriptive data for donor-donor (DD) and donor-infertile (DI) cohorts

The present work aimed to determine the impact of sperm DNA breaks on ICSI outcomes when donor or patient oocytes are used. For this purpose, whether populations of semen donors of each cohort (i.e., oocyte donation, DD; or cycles with the own couple’s oocytes, DI) were comparable was first examined. As shown in Table 1, no significant differences between men populations were observed for any of the assessed sperm parameters (*P* > 0.05). Regarding females, the age of the patient (i.e., woman receiving embryos) was greater in the donor-donor than in the donor-infertile cohort (*P* < 0.0001), but women providing oocytes were younger in the donor-donor cohort (*P* < 0.0001). No differences in weight or body mass index of females providing oocytes or receiving embryos were observed between cohorts (*P* > 0.05).

**Table 1:** Sperm quality parameters in donor samples used in the cycles involving oocyte donors (donor-donor cohort) and in those using oocytes from patients (donor-infertile cohort)

	Donor-donor cohort		Donor-infertile cohort		*P*-value
	Mean ± SD	95% CI	Mean ± SD	95% CI	
Fresh semen					
Total sperm count (× 106 sperm)	294.1 ± 125.8	[245.3 to 342.9]	245.2 ± 143.1	[195.3 to 295.2]	0.163
Sperm concentration (× 106 sperm / mL)	85.39 ± 31.47	[73.19 to 97.59]	81.82 ± 35.57	[68.80 to 93.62]	0.484
Progressive motility (%A + B)	60.73 ± 11.73	[56.18 to 65.27]	60.93 ± 12.89	[56.43 to 65.43]	0.924
Non-progressive motility (%C)	7.45 ± 4.63	[5.66 to 9.25]	7.84 ± 4.56	[6.25 to 9.43]	0.706
Immotile sperm (% D)	31.82 ± 10.09	[27.91 to 35.74]	31.23 ± 12.19	[26.98 to 35.49]	0.595
Motile sperm selection after thawing					
Concentration (M/mL)	34.42 ± 17.83	[27.50 to 41.33]	37.22 ± 19.67	[30.36 to 44.08]	0.557
Progressive motility (%A + B)	35.84 ± 10.99	[31.58 to 40.11]	37.25 ± 8.599	[34.25 to 40.25]	0.610
Non-progressive motility (%C)	6.75 ± 3.59	[5.35 to 8.14]	7.803 ± 4.383	[6.27 to 9.33]	0.444
Immotile sperm (% D)	57.41 ± 12.55	[52.54 to 62.27]	54.94 ± 10.40	[51.31 to 58.57]	0.441
Morphology					
Abnormal shapes (%)	84.32 ± 3.56	[82.94 to 85.70]	83.79 ± 3.914	[82.43 to 85.16]	0.785
Head Anomalies (%)	55.43 ± 9.267	[51.84 to 59.02]	53.65 ± 9.422	[50.36 to 56.93]	0.524
Flagellum abnormalities (%)	1.82 ± 1.16	[1.37 to 2.27]	1.65 ± 1.13	[1.26 to 2.04]	0.519
Mixed Abnormalities (%)	27.07 ± 9.100	[23.54 to 30.60]	28.47 ± 10.54	[24.79 to 32.15]	0.675

ICSI and clinical outcomes for both cohorts are shown in Table 2. While the number of fertilized oocytes, the number of blastocysts and the number of embryos transferred for each donor (*P* > 0.05) did not differ between cohorts, blastocyst rates and percentages of blastocysts obtained per oocyte injected were higher in the donor-donor than in the donor-infertile cohort (*P* = 0.021 and *P* = 0.006). Although rates of pregnancy per transfer and those of cumulative pregnancy per cycle tended to be lower in the donor-infertile than in the donor-donor cohort, the differences were not statistically significant (*P* = 0.093 and *P* = 0.147, respectively). Live-birth rates per embryo transfer were lower in the donor-infertile than in the donor-donor cohort (*P* = 0.041), but rates of cumulative live birth per cycle did not reach statistically significant differences *P* = 0.064). Miscarriage rates were similar between the two cohorts (*P* = 0.242).

**Table 2:** Data for ART cycles in each cohort (donor-donor or donor-infertile). (A) Female physiological parameters and (B) embryo development

	Donor-donor cohort		Donor-infertile cohort		*P*-value
	Average ± SD	95% CI	Average ± SD	95% CI	
A					
Age of female patient (years)	43.25 ± 4.019	[42.13 to 44.37]	36.91 ± 5.548	[35.45 to 38.37]	** < 0.0001**
Weight of female patient (kg)	62.5 ± 11.57	[59.28 to 65.72]	58.96 ± 17.3	[54.41 to 63.51]	0.418
BMI of female patient	22.91 ± 3.549	[21.92 to 23.9]	21.14 ± 6.013	[19.56 to 22.72]	0.107
Age of the woman who provides oocytes (years)	25.17 ± 4.057	[24.04 to 26.3]	36.91 ± 5.548	[35.45 to 38.37]	** < 0.0001**
Weight of the woman who provides oocytes (kg)	60.52 ± 9.948	[57.75 to 63.29]	58.96 ± 17.3	[54.41 to 63.51]	0.789
BMI of the woman who provides oocytes	22.2 ± 3.138	[21.33 to 23.08]	21.14 ± 6.013	[19.56 to 22.72]	0.557
B					
Average number of MII oocytes recovered	16.15 ± 13.62	[10.76 to 21.54]	15.65 ± 11.65	[11.58 to 19.71]	0.865
Average number of fertilized embryos	11.78 ± 9.79	[7.91 to 15.65]	11.21 ± 8.94	[8.09 to 14.33]	0.928
Average number of blastocysts	7.26 ± 5.74	[4.99 to 9.53]	5.62 ± 5.25	[3.79 to 7.45]	0.138
Average number of embryos transferred per donor	2.11 ± 1.85	[1.38 to 2.84]	2.09 ± 1.90	[1.43 to 2.75]	> 0.999
Fertilization rate (%)	75.76 ± 17.62	[68.92 to 82.59]	71.89 ± 14.77	[66.65 to 77.13]	0.275
Blastocyst rate (%)	61.55 ± 19.65	[53.93 to 69.17]	50.38 ± 16.89	[44.39 to 56.37]	**0.021**^a^
Blastocysts/MII oocytes injected (%)	48.27 ± 22.06	[39.71 to 56.82]	35.63 ± 12.57	[31.17 to 40.09]	**0.006**^a^
Pregnancy/embryo transfer (%)	61.40% (35/57)		46.47% (33/71)		0.093
Cumulative pregnancy/cycle (%)	71.42% (35/49)		57.89% (33/57)		0.147
Live birth/embryo transfer (%)	56.14% (32/57)		38.02% (27/71)		**0.041**^a^
Cumulative live birth/cycle (%)	65.30% (32/49)		47.37% (27/57)		0.064
Miscarriage rate/pregnancy (%)	8.57% (3/35)		18.18% (6/33)		0.242

Bold indicates statistically significant *P*-values

*A* corresponds to female physiological parameters, *B* corresponds to embryo development parameters

### Basic seminal parameters in male donors do not have an impact on ICSI outcomes, regardless of the oocyte origin

**Fig. 1:**
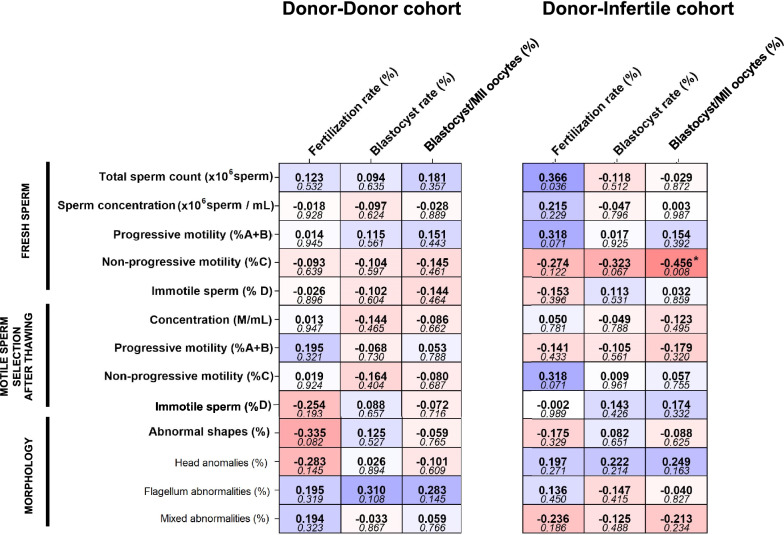
Correlation coefficients (upper value) and *P*-values (bottom value) between basic seminal parameters and ICSI outcomes in cycles involving oocyte donors (donor-donor cohort) and in cycles using oocytes from patients (donor-infertile cohort). (*) indicates statistically significant correlations (*P* ≤ 0.05)

As shown in Fig. 1, no correlation between most conventional semen variables (sperm count, motility and morphology) and ICSI outcomes (fertilization and blastocyst rates) was observed in any of the cohorts studied (*P* > 0.05). Only a correlation between the percentage of non-progressively motile sperm and the percentage of blastocysts in the donor-infertile cohort was found (*P* < 0.05).

### Overall DNA damage in sperm is correlated to fertilization rates and the percentage of blastocysts only in cycles with donor oocyte

**Fig. 2:**
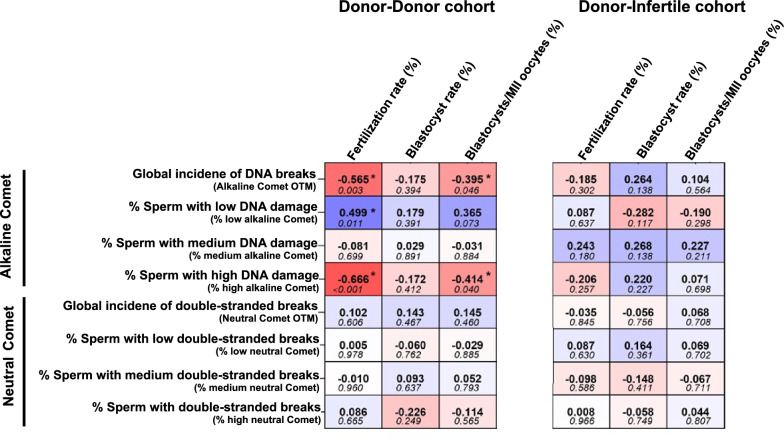
Correlation coefficients (upper value) and *P*-values (bottom value) between different types of sperm DNA damage and ICSI outcomes in cycles involving oocyte donors (donor-donor cohort) and in cycles using oocytes from patients (donor-infertile cohort). (*) indicates statistically significant correlations (*P* ≤ 0.05)

As routine basic semen parameters showed no effect on embryo outcomes, whether the incidences of overall and double-stranded sperm DNA breaks influence embryo development was tested. It was first confirmed that neither the incidence of overall sperm DNA breaks nor that of the double-stranded ones differed between the two men populations involved in each cohort (i.e., double donation cycles and cycles including oocytes from infertile couples; Table 3). Afterwards, correlations between sperm DNA damage and ICSI outcomes were calculated (Fig. 2). In the donor-donor cohort, the incidence of overall sperm DNA breaks (OTM) was found to be negatively correlated to fertilization rate (Rs = − 0.565; *P* = 0.003) and with the percentage of blastocysts over the number of injected MII-oocytes (Rs = − 0.395; *P* = 0.046). Similarly, the percentage of sperm with high overall DNA damage was observed to be negatively correlated to fertilization rate (Rs = − 0.666; *P* < 0.001) and to the percentage of blastocysts over the number of injected MII-oocytes (Rs = − 0.414; *P* = 0.040). Remarkably, the aforementioned correlations were not seen in the donor-infertile cohort (*P* > 0.05).

**Table 3:** Incidence of overall DNA damage (alkaline Comet) and of double-stranded breaks (neutral Comet) in donor sperm, and percentages of sperm with low, medium or high DNA fragmentation

	Donor-donor cohort		Donor-infertile cohort		*P*-value
	Average ± SD	95% CI	Average ± SD	95% CI	
Global incidence of DNA breaks (Alkaline comet OTM)	32.57 ± 6.86	[29.80–35.34]	32.89 ± 7.62	[30.23–35.55]	0.915
Percentage of sperm with low incidence of DNA damage (% low alkaline Comet)	32.71 ± 23.70	[22.93–42.49]	32.73 ± 24.30	[24.11–41.35]	0.999
Percentage of sperm with medium incidence of DNA damage (% medium alkaline Comet)	47.05 + 16.22	[40.35–53.74]	46.12 ± 15.98	[40.45–51.79]	0.916
Percentage of sperm with high incidence of DNA damage (%high alkaline Comet)	20.24 ± 18.15	[12.75–27.74]	21.18 ± 18.45	[14.64–27.72]	0.928
Incidence of double-stranded DNA breaks (Neutral comet OTM)	3.29 ± 2.27	[2.41–4.17]	3.32 ± 1.89	[2.66–3.98]	0.471
Percentage of sperm with low incidence of double-stranded DNA breaks (% low neutral Comet)	89.37 ± 17.37	[82.64–96.11]	89.44 ± 15.11	[94.17–94.71]	0.533
Percentage of sperm with medium incidence of double-stranded DNA breaks (%medium neutral Comet)	10.15 ± 16.50	[3.75–16.55]	10.18 ± 14.52	[5.11–15.24]	0.533
Percentage of sperm with high incidence of double-stranded DNA breaks (%high neutral Comet)	0.48 ± 1.11	[0.051–0.91]	0.44 ± 0.79	[0.17–0.72]	0.850

### Overall DNA damage in sperm delays embryo development in the donor-donor cohort

**Fig. 3:**
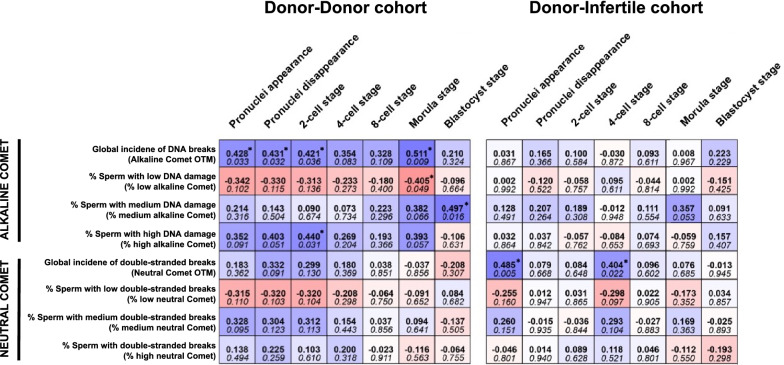
Correlation coefficients (upper value) and *P*-value (bottom value) between sperm DNA damage and the time required for the embryo to reach a specific development stage. (*) indicates statistically significant correlations (*P* ≤ 0.05)

Having found that overall DNA damage in sperm was correlated to ICSI outcomes in the donor-donor but not in the donor-infertile cohort, and because the two cohorts differed in blastocyst but not in fertilization rates, we surmised that, while poor-quality oocytes could fail to repair sperm DNA damage and thus become arrested, the good-quality ones would repair these sperm DNA breaks. This would cause a delay but embryos would continue to develop. To test this hypothesis, embryo kinetics through time-lapse were determined and correlated with variables assessing sperm DNA damage (Fig. 3).

In the donor-donor cohort, the incidence of overall DNA breaks in sperm (OTM) was found to be correlated with the time required for the formation of pronuclei, the time required for the disappearance of pronuclei, and the time spent to reach the 2-cell (t2) and morula stages (*P* < 0.05, Fig. 3). In addition, the incidence of overall DNA breaks in sperm was observed to be correlated to the time elapsed between the 8-cell and morula stages (Rs = 0.424; *P* = 0.034). Moreover, the proportion of sperm with an intermediate overall DNA damage was found to be correlated to the time needed for the embryo to reach the blastocyst stage (Rs = 0.497; *P* = 0.01), and the proportion of sperm with high overall DNA fragmentation was seen to correlate to the time required for the embryo to reach the 2-cell stage (Rs = 0.440; *P* = 0.031). In contrast, neither the incidence of double-stranded DNA breaks (OTM) nor the percentage of sperm with double-stranded DNA damage correlated with embryo kinetics (*P* > 0.05, Fig. 3).

In the donor-infertile cohort, no parameter evaluating overall DNA damage in sperm was found to be correlated to embryo kinetics (*P* > 0.05, Fig. 3). The incidence of double-stranded DNA breaks, however, correlated to the time between ICSI and the formation of pronuclei (Rs = 0.485; *P* = 0.005) and that needed for the embryo to reach the 4-cell stage (Rs = 0.404; *P* = 0.022).

### A high incidence of sperm DNA breaks leads to earlier embryo development arrest

In previous analyses, sperm DNA damage was observed to delay embryo development. This, nonetheless, does not necessarily entail an arrest of that development. To investigate this further, whether the incidence of sperm DNA breaks or the percentage of sperm with fragmented DNA were correlated to the time that embryo arrest occurred was tested in the two cohorts.

On the one hand, the incidence of sperm DNA breaks, either overall or double-stranded, was not found to be correlated to the day that embryo arrest occurred, in any of the two cohorts (*P* > 0.05). Yet, a tendency close to statistical significance was observed for the correlation between the incidence of overall DNA breaks in sperm and the day of embryo arrest in the donor-donor cohort (Rs = − 0.386; *P* = 0.062).

**Fig. 4:**
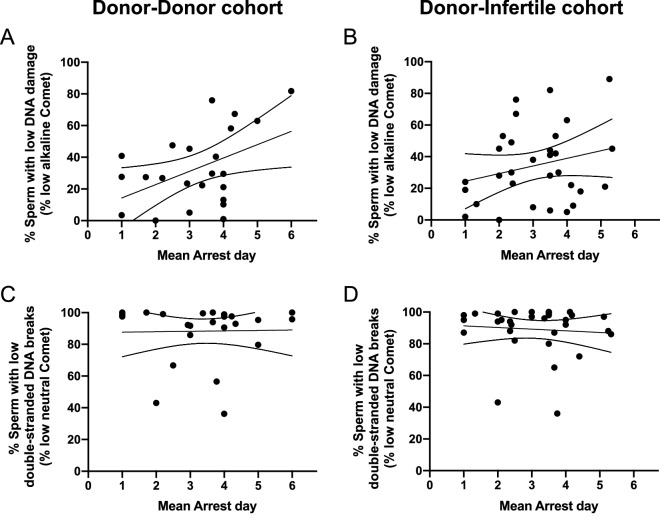
Correlations of the day when embryo development arrested with the percentage of sperm with low overall DNA damage (**A**, **B**), and with the percentage of sperm with low double-stranded DNA breaks (**C**, **D**)

On the other hand, the percentage of sperm with low or inexistent overall DNA damage correlated to the day of embryo arrest in the donor-donor cohort (Rs = 0.458; *P* = 0.028; Fig. 4A), but not in the donor-infertile one (Rs = 0.244; *P* = 0.185, Fig. 4B). Finally, the percentage of sperm with double-stranded DNA breaks did not correlate with the day of embryo arrest in any of the two cohorts (*P* > 0.05, Fig. 4C, D).

## Discussion

Previous research showed that sperm DNA breaks have an adverse effect on natural reproduction [51]. Furthermore, the most recent meta-analysis assessing the impact of paternal genotoxic damage on ART outcomes supported that alterations in sperm DNA exert a detrimental impact on conventional IVF. Yet, this meta-analysis also evidenced a lack of consensus in the case of ICSI, as only 50–60% of the studies reported a negative effect of sperm DNA damage on laboratory and clinical outcomes [25]. Gaining further knowledge on this topic is of outmost importance because understanding the factors reducing ICSI effectiveness is likely to help while providing advice on the best treatment to couples with implantation failures. As described in the Introduction, however, ICSI outcomes in infertile couples may be influenced and biased by many factors. For these reasons, the influence of sperm DNA integrity on ICSI outcomes in the present work was evaluated using semen and oocytes from healthy young donors (donor-donor cohort); the results were compared with those of cycles in which oocytes came from patients (donor-infertile cohort).

To the best of the authors’ knowledge, this is the first study investigating the effects of sperm DNA damage on ICSI outcomes in a donor-donor cohort (i.e., double donation cycles), providing a new angle to address the issue. As the influence of sperm DNA integrity on ICSI outcomes was interrogated in the two cohorts, how the origin of oocytes shapes that influence could be addressed and confounding female factors could be avoided. The main conclusion of this approach was that sperm DNA integrity does have an impact on ICSI outcomes, particularly when donor, good-quality oocytes are used. In contrast and as data from the donor-infertile cohort (i.e., oocytes from infertile women were fertilized) showed, oocyte quality could have a major impact on embryo development and thus mask the effects of sperm DNA damage. Interestingly, embryo kinetics data from the donor-donor cohort indicated that the incidence of overall DNA breaks in sperm has an influence on the times elapsed for pronuclear formation and disappearance, the interval needed for the fertilized oocyte to reach the 2-cell stage, and the length of the transition from the 8-cell to morula stages. These effects were not observed when oocytes from infertile women were involved (donor-infertile cohort), as only the incidence of double-stranded DNA breaks in sperm was related to embryo kinetics, thus suggesting that this type of breaks may have a greater impact in development.

Conventional spermiogram parameters were not detected to be associated to fertilization or blastocyst rates, in line with what reported previously in double donation studies [52], research involving infertile men and oocyte donors [53], and studies including infertile males and females [54–56]. The lack of association between these sperm variables and ICSI outcomes demands clinicians and researchers to intensify investigations into molecular biomarkers that may be used to predict ART success and understand the complex multifactorial etiology of infertility. In the present study, both the incidence of overall DNA breaks in sperm and the percentage of sperm with high DNA damage were noticed to correlate to fertilization and blastocyst rates when the two gametes came from healthy donors (i.e., donor-donor cohort). The same results, nevertheless, were not observed when oocytes from infertile patients were involved (i.e., donor-infertile cohort), even though blastocyst rates were lower. Because the only difference between the two cohorts was the origin of the oocytes, these data clearly suggest that the impact of sperm DNA integrity on ICSI outcomes relies upon oocyte quality. Hence, one may thus reasonably posit that the differences in the origin of oocytes could be one of the reasons to explain the inconsistencies in the literature about the actual influence of sperm DNA integrity on ICSI outcomes, particularly when infertile patients are committed [57, 58]. In effect, while a previous work including infertile males showed that the male factor had a great impact on pregnancy outcomes when oocytes from infertile or older women were used [14, 59] and another study using donor oocytes reported an association between sperm genotoxic damage and ICSI outcomes [60], other researchers concluded that sperm DNA damage in infertile males was not associated to ICSI outcomes, regardless of the oocyte origin [17]. Whilst these three opposite results clearly reflect the controversy on the topic, one has to note that all them were conducted in infertile males; yet, the current study is the first to approach the matter in a double donation cohort. Indeed, the fact that the influence of different types of sperm DNA breaks (single- and double-stranded) on ICSI outcomes was evaluated with respect to the oocyte origin (own or donated) is one of the strengths of this study, as it reduces the risk of bias due to unknown alterations in male or female gametes from infertile individuals [29, 30, 35]. Moreover, as this work involved donor sperm, it is worth mentioning that although three different grades of sperm DNA damage could be established, figures fell into the normal range and, thus, infertile individuals could present greater sperm DNA fragmentation [10].

A remarkable finding of this study was that not only were ICSI outcomes poorer when oocyte from patients were involved, but correlations between sperm DNA damage and those outcomes were lost. These findings suggest that the effects of sperm DNA damage on ICSI outcomes may be hidden when oocyte quality is diminished, and thus support that oocyte alterations have a larger influence on embryo development than sperm DNA breaks do. This is in agreement with previous research reporting that oocyte quality is one of the main causes of embryo aneuploidy and implantation failure [29, 61, 62]. Conversely, when oocytes from greater quality—because they come from donors—were involved, ICSI outcomes were better and it was thus easier to determine the influence of sperm DNA integrity on embryo development. This would not obviously exclude that sperm DNA fragmentation could worsen ICSI outcomes if gametes from male and female patients are combined, as suggested by previous studies [13, 59, 63].

The fact that sperm DNA fragmentation influences ICSI outcomes suggests that embryos activate DNA damage repair (DDR) mechanisms when they have to deal with broken paternal DNA. Previous works demonstrated that the canonical DNA damage repair pathways present in somatic cells may also be active in oocytes and embryos [4, 5]. Thanks to these mechanisms, DNA mismatches, excision of bases (BER), single-stranded breaks and double-stranded breaks (non-homologous end joining, NHEJ; and homologous recombination, HR) can be repaired [64]. The activation of these mechanisms appears to slow down DNA replication and embryo development [65–67]. Herein, whether sperm DNA breaks in donors could bring to the activation of these mechanisms and delay embryo development was tested through embryo time-lapse assessment. Interestingly, positive correlations of the incidence of overall DNA damage in sperm with the time required for the formation of pronuclei, their disappearance, and the time spent until the embryo divided for the first time were observed. As sperm cells contain one of the first enzymes of the BER pathway, 8-oxoguanine DNA glycosylase (OGG1) [68], zygotes could follow this route to repair chromatin oxidative damage, thus delaying the initial stages of embryo development [69]. In addition, because protamine-condensed paternal chromatin is inherited along with the nuclear matrix, and previous works suggested that double-strand break ends may be attached to the nuclear matrix [70], the zygote would have the opportunity to repair paternal DNA breaks at very early development stages through NHEJ or HR [6]. Moreover, the activation of BER, HR and NHEJ mechanisms could explain the delay in the formation of zygotes and in their further development, while leading to high blastocyst rates. According to the results of this study, after embryos underwent the first division, there was no correlation between sperm DNA damage and the kinetics of the second and third division. This contrasts with the positive correlation observed between the incidence of overall DNA damage in donor sperm and the time elapsed for the transition of the 8-cell to the morula stage. Similarly, a delay in embryo development was observed in infertile couples when sperm with a high incidence of double-strand breaks were used [42]. Herein, a combination of single- and double-stranded breaks appeared to have similar effects on embryo development in the donor-donor cohort, which could be related to the higher capacity of the DDR mechanism in oocytes from healthy individuals [71, 72]. It is worth emphasizing that, while this is the first study assessing the relationship between sperm DNA damage and ICSI outcomes in a donor-donor cohort, the results obtained herein concur with previous research using sperm from infertile patients and donor oocytes, where slower embryo development was observed when sperm DNA fragmentation was high [73–75].

The present work also showed that the occurrence of embryo arrest was correlated with the percentage of sperm with high DNA damage in the donor-donor cohort. Related to this, earlier research demonstrated that sperm with extensive DNA damage may drive to embryo arrest following ICSI, probably because DNA replication is impaired due to the activation of the S-phase or spindle-assembly checkpoints [65, 76]. In addition, at early blastocyst stages, apoptosis may be triggered in response to sperm DNA damage, thus arresting those embryos that present a high degree of chromosome fragmentation or unrepaired DNA [66, 77].

Finally, regarding clinical outcomes, this study demonstrated that while pregnancy/embryo transfer and cumulative pregnancy/cycle rates were not significantly different between cohorts, live birth rates per embryo transfer were statistically lower in the donor-infertile cohort than in the donor-donor one. This observation is consistent with the greater effect of the female counterpart on postimplantational events, and agrees with previous results published in the literature [32, 78, 79].

## Conclusions

In conclusion, this study, which is the first involving a donor-donor gamete cohort, demonstrated that sperm DNA integrity does have an influence on fertilization and blastocyst rates after ICSI. In addition, the incidence of DNA breaks in donor sperm was found to be associated to a delayed embryo development, suggesting that the DDR mechanism is activated at zygote and morula stages. Because these relationships were not observed when oocytes from patients were involved, the possibility that female factors mask the paternal effects in ICSI cycles is very likely.

## Material and methods

### Participants, semen samples and ethics

Semen samples coming from 61 healthy male donors were provided for ICSI cycles under a semen donation program. These samples were split into two groups: (a) 27 were used in 49 ICSI cycles, microinjecting oocytes from healthy young donors (donor-donor cohort), and the other 34 were utilized in 57 ICSI cycles involving oocytes from infertile patients diagnosed with primary infertility (donor-infertile cohort). Inclusion and exclusion criteria for semen donors were: good overall health, normal karyotype, lack of urinary tract infections, lack of previous history of hereditary or sexually transmitted diseases, and good seminal quality. Detailed criteria for inclusion/exclusion of semen donors in the present study are shown in Additional file 1: Table S1, and values for the available male donor characteristics are summarized in Additional file 1: Table S2.

For oocyte donors, general inclusion criteria were being less than 34 years old, having a normal karyotype, lack of history of hereditary or sexually transmitted diseases, not suffering from anatomical or endocrine alterations, and not having a low ovarian reserve. A more detailed list of the screenings conducted before the inclusion of oocyte donors can be found in Additional file 1: Table S1.

Men collected semen samples by masturbation into a sterile cup after two to five days of sexual abstinence. Immediately after ejaculation, samples were brought to the laboratory, where they were allowed to liquefy for 30 min at 37 °C. Afterwards, an aliquot of the sample was designed to assess basic semen parameters, including pH, volume, concentration, sperm count, motility and morphology. The remaining volume was cryopreserved following the protocol described below; cryopreserved samples were intended to the evaluation of sperm DNA integrity and were used for ICSI.

This investigation complied with the Spanish legislation and the Helsinki Declaration for Biomedical Research. All donors signed an informed consent; the study was approved by the Ethics Committee of Hospital Doctor Josep Trueta, Catalan Health Institute (Girona, Spain; ref. PTI-HUMA 10012018).

### Evaluation of sperm concentration, motility and morphology

After samples liquefied completely, a basic sperm analysis was conducted following the instructions of the 5th Edition of WHO Laboratory Manual for the Examination and Processing of Human Semen [80]. First, macroscopical parameters (volume, viscosity and pH) were evaluated. Volume was assessed through a volumetric flask, viscosity (normal or viscous) was determined using a Pasteur pipette, and pH was measured with a pH test strip (range 6.0 to 10.0), which was compared to a calibration one. Regarding microscopical evaluation, sperm motility was estimated using an automated sperm analysis system (LensHooke^®^ X1 PRO [X1 PRO], Bonraybio, Taichung, Taiwan), following the manufacturer’s instructions. Concentration was recorded as sperm count/mL. For motility, percentages of sperm cells with progressive motility (% type A + % type B) and non-progressive motility (% type C), and those of immotile sperm (% type D) were evaluated. Sperm morphology was assessed through Diff-Quick staining (RAL Diagnostics, Martillac, France) following the manufacturer’s protocol. Percentages of morphologically abnormal sperm were determined under a bright-field microscope at 1000 × . Abnormalities in the flagellum, alterations in the sperm head and mixed abnormalities were also considered.

### Cryopreservation and thawing of sperm samples

Cryopreservation was conducted following a routinely-established protocol at the semen bank. Briefly, sperm were slowly mixed with Freezing Medium (Fujifilm Irvine Scientific, Santa Ana, CA, USA), which contains TEST-yolk buffer, at a 1:1 (v:v) ratio. After that, the mixture was packaged into labelled straws, which were suspended in nitrogen vapors for 20 min. Immediately after, straws were plunged into liquid nitrogen and stored until required.

For thawing, straws were incubated at room temperature for 40 s, and at 30 °C for further 40 s. Following this, the two straw ends were cut with sterile scissors, and sperm were transferred into sterile tubes. Next, samples were slowly diluted in PureSperm Wash medium (PureSperm, Nidacon, Sweden) at 37 °C, centrifuged at 300 g and room temperature for 5 min, washed again in the same medium tempered, and adjusted to a concentration of 2 × 10^6^ sperm/mL.

### Comet assay

Alkaline and neutral Comet assays were carried out to evaluate the incidence of single- and double-stranded DNA breaks, respectively. The protocol was based on that described before by Casanovas et al. [42], with some modifications in data analysis.

#### Preparation of sperm-agarose slides and lysis of samples

Sperm concentration was adjusted to 1 × 10^6^ sperm/mL. Thereafter, samples were mixed with 1% low melting point agarose (37 ºC) at a 1:2 (v:v) ratio, reaching an agarose concentration of 0.66%. Subsequently, 6.5 µL of the mixture was placed onto 1% agarose pre-treated slides for gel adhesion, and covered with a coverslip. Agarose-sample mixture was allowed to jellify at 4 ºC for 5 min on the top of a metal plate; next, coverslips were gently removed. Two slides were prepared, one for the alkaline Comet and one for the neutral Comet. Both slides were immersed into the first lysis solution containing 0.8 M Tris–HCl, 0.8 M DTT and 1% SDS (pH = 7.5) for 30 min, and then in the second lysis solution containing 0.4 M Tris–HCl, 0.4 M DTT, 50 mM EDTA, 2 M NaCl and 1% Tween20 (pH = 7.5) for further 30 min. After these two incubations, slides were washed in distilled water for 2 min.

#### Electrophoresis and dehydration

Different protocols were followed for each Comet analysis. For the alkaline Comet, slides were incubated in a cold alkaline solution (4 °C) containing 0.03 M NaOH and 1 M NaCl for 5 min, and electrophoresed in an alkaline buffer (0.03 M NaOH; pH = 13) at 1 v/cm for 4 min. For the neutral Comet, slides were electrophoresed in a TBE buffer (0.445 M Tris–HCl, 0.445 M Boric acid and 0.01 M EDTA; pH = 8) at 1 v/cm for 12.5 min, and subsequently incubated in a 0.9% NaCl solution for 2.5 min. After electrophoresis, both slides were submerged into a neutralization solution containing 0.4 M Tris–HCl (pH = 7.5) for 5 min, and dehydrated in an ethanol series (70%, 90%, and 100%; 2 min each step). Finally, slides were dried horizontally.

#### Staining and imaging

Comets were stained by incubation of dried slides with 1 × SYTOX orange (Invitrogen, Waltham, MA, USA) at room temperature for 15 min. Slides were subsequently washed in distilled water for 2 min and allowed them to dry horizontally. Samples were observed under a Zeiss Imager Z1 epifluorescence microscope (Carl Zeiss AG, Oberkochen, Germany) at 100 × magnification, and comets were captured using the Axiovision 4.6 software (Carl Zeiss AG, Oberkochen, Germany), avoiding the overexposure of comet heads and tails.

#### Comet analysis

The open-access CometScore v2.0 software (RexHoover) was used for the analysis of individual comets. First, background was adjusted in each image, and individual comets were examined using the automatic analysis option. After that, a manual revision of the analysis was conducted in order to eliminate captures not corresponding to comets, to remove overlapping comets, and to correct the comet head/tail detection. At least 100 correctly analyzed comets were required for each sample; when this figure was not reached, more pictures were taken and the process was repeated.

The CometScore software provides a wide variety of parameters defining different aspects of the DNA present in a given cell. Among them, the Olive Tail Moment (OTM) was chosen as a quantitative measurement for the incidence of DNA breaks, as different reports suggest that it is the most informative parameter for this purpose [39, 81]. Olive tail moment is calculated as (tail mean intensity—head mean intensity) × %tail DNA /100. Tail DNA, computed as tail intensity divided by comet intensity, was also recorded.

In order to determine the percentage of sperm with high DNA fragmentation, a principal component analysis (PCA) was conducted using OTM and Tail DNA parameters. First, these parameters were sorted into one PCA component, and the regression scores for this variable were used to classify each comet through a cluster analysis, using the between-groups linkage method based on the Euclidean distance. For each comet variant, three sperm populations were obtained, which were defined as with low, medium or high DNA damage. The percentages of sperm in each of these three categories were recorded in every sample.

### Assisted reproduction procedures

#### Sperm preparation

Thawed sperm were washed through a density gradient (90–75%; PureSperm, Nidacon, Sweden). Immediately after, selected motile sperm were washed twice by centrifugation at 200 g and room temperature for 20 min. The resulting pellet was resuspended in 2 mL of gamete medium (Sequential Fert., ORIGIO, Denmark), and sperm concentration was adjusted to 0.1–0.5 × 10^6^ progressively motile sperm/mL before ICSI.

#### Ovarian stimulation, oocyte retrieval and denudation

Pituitary down-regulation was carried out by administering Gonadotrophin Releasing Hormone (GnRH) agonist or antagonist. In order to achieve ovarian stimulation, an injectable recombinant or urine-derived Follicle Stimulating Hormone (FSH) was used. The final follicular maturation was triggered with human Chorionic Gonadotrophin (hCG) and/or GnRH, when the two leading follicles measured ≥ 17 mm in diameter.

Oocyte-cumulus complexes (COCs) were harvested through an ultrasound guide, which was conducted after 36 h of triggering. COCs were cultured in fertilization media (Sequential Fert, ORIGIO, Denmark), covered with LifeGuard oil (LifeGlobal, Cooper-Surgical, Denmark), and prepared for ICSI. For this purpose, oocytes were mechanically denuded in the presence of hyaluronidase (FertiPro, Belgium).

#### ICSI and embryo culture

Before ICSI, sperm were diluted in FertiCult Flushing medium (FertiPro, Beernem, Belgium) containing 10% polyvinylpyrrolidone (PVP). Sperm with progressive motility were immobilized, and ICSI was carried out in metaphase II oocytes, between 39 and 41 h after hCG administration. Putative embryos were cultured in EmbryoSlide culture dish microwells (Vitrolife, Göteborg, Sweden) containing a single culture medium (SAGE 1-Step with Human Albumin Solution, ORIGIO, Måløv, Denmark). Uninterrupted culture was conducted in an EmbryoScope incubator (Vitrolife, Göteborg, Sweden) at 37 ºC, 7% O_2_, balanced N_2_ and 6% CO_2_.

#### Time-lapse monitoring and embryo scoring

The time-lapse incubator captured images from seven different focal-planes of embryos every 15 min between ICSI and day 6. Image sequences were compiled, and analysis of embryo kinetics was conducted using the EmbryoViewer software (Vitrolife, Göteborg, Sweden). Experienced embryologists scored embryos with the assistance of the KIDScore D5 algorithm (Vitrolife, Denmark), which recorded morphokinetics. Blastocysts were evaluated using the Gardner criteria [82].

#### Vitrification/warming of blastocysts and embryo transfer

Blastocysts with a quality equal to or larger than 3BB were vitrified with Kitazato vitrification medium (Kitazato, Tokyo, Japan) and Cryotop® (Kitazato). Embryo transfer was performed in a freeze-all basis. In brief, embryos were first warmed at 37 °C in Kitazato medium (Kitazato), and a single embryo at day 5–6 was subsequently transferred into the uterine cavity. After 12–15 days, gestation was tested using a β-hCG pregnancy test and, when positive, pregnancies were followed by ultrasound.

### Statistical analysis

Statistical analyses were conducted with Statistical Package for Social Sciences ver. 27 (IBM Corp., Armonk, NY, USA) and graphs were plotted with GraphPad Prism ver. 8 (La Jolla, CA, USA). First, normal distribution was examined with the Shapiro–Wilk test, and homoscedasticity was checked with the Levene test. As variables did not fit with parametric assumptions, non-parametric tests were used. Correlations, therefore, were evaluated through the Spearman test. For comparisons between multiple variables, Kruskal–Wallis, Mann–Whitney and Dunn’s post-hoc tests were run as an alternative to ANOVA. The Chi-squared test was also utilized when clinical outcomes (pregnancy and live birth rates) were compared between two cohorts. A *P*-value ≤ 0.05 was established as a requirement to consider the values statistically significant.

## Supplementary Information

**Additional file 1: Table S1.** Exclusion criteria for semen and oocyte donors. **Table S2.** Characteristics of sperm donors.

## Data Availability

Sets of data collected during the current study are available from the corresponding author on reasonable request.

## References

[1] Oliva R (2006). Protamines and male infertility. Hum Reprod Update.

[2] Sotolongo B, Huang TTF, Isenberger E, Ward WS (2005). An endogenous nuclease in hamster, mouse, and human spermatozoa cleaves DNA into loop-sized fragments. J Androl.

[3] Sakkas D, Alvarez J (2010). Sperm DNA fragmentation: mechanisms of origin, impact on reproductive outcome, and analysis. Fertil Steril Fertil Steril.

[4] Jaroudi S, Kakourou G, Cawood S, Doshi A, Ranieri DM, Serhal P (2009). Expression profiling of DNA repair genes in human oocytes and blastocysts using microarrays. Human Reproduction.

[5] Setti AS, de Braga DP, AF, Provenza RR, Iaconelli A, Borges E. (2021). Oocyte ability to repair sperm DNA fragmentation: the impact of maternal age on intracytoplasmic sperm injection outcomes. Fertil Steril.

[6] Derijck A, van der Heijden G, Giele M, Philippens M, de Boer P (2008). DNA double-strand break repair in parental chromatin of mouse zygotes, the first cell cycle as an origin of de novo mutation. Hum Mol Genet.

[7] Rich T, Allen RL, Wyllie AH (2000). Defying death after DNA damage. Nature Nature.

[8] Lewis SEM, Simon L (2010). Clinical implications of sperm DNA damage. Hum Fertil.

[9] Saleh RA, Agarwal A, Nelson DR, Nada EA, El-Tonsy MH, Alvarez JG (2002). Increased sperm nuclear DNA damage in normozoospermic infertile men: a prospective study. Fertil Steril.

[10] Ribas-Maynou J, García-Peiró A, Abad C, Amengual MJ, Navarro J, Benet J (2012). Alkaline and neutral comet assay profiles of sperm DNA damage in clinical groups. Hum Reprod.

[11] Garolla A, Cosci I, Bertoldo A, Sartini B, Boudjema E, Foresta C (2015). DNA double strand breaks in human spermatozoa can be predictive for assisted reproductive outcome. Reprod Biomed Online.

[12] Simon L, Brunborg G, Stevenson M, Lutton D, McManus J, Lewis SEM (2010). Clinical significance of sperm DNA damage in assisted reproduction outcome. Hum Reprod.

[13] Simon L, Proutski I, Stevenson M, Jennings D, McManus J, Lutton D (2013). Sperm DNA damage has a negative association with live-birth rates after IVF. Reprod Biomed Online.

[14] Meseguer M, Santiso R, Garrido N, García-Herrero S, Remohí J, Fernandez JL (2011). Effect of sperm DNA fragmentation on pregnancy outcome depends on oocyte quality. Fertil Steril.

[15] Henkel R, Kierspel E, Hajimohammad M, Stalf T, Hoogendijk C, Mehnert C (2003). DNA fragmentation of spermatozoa and assisted reproduction technology. Reprod Biomed Online.

[16] Sivanarayana T, Ravi Krishna C, Jaya Prakash G, Krishna KM, Madan K, Sudhakar G (2014). Sperm DNA fragmentation assay by sperm chromatin dispersion (SCD): Correlation between DNA fragmentation and outcome of intracytoplasmic sperm injection. Reprod Med Biol.

[17] Esbert M, Pacheco A, Vidal F, Florensa M, Riqueros M, Ballesteros A (2011). Impact of sperm DNA fragmentation on the outcome of IVF with own or donated oocytes. Reprod Biomed Online.

[18] Thomson LK, Zieschang J-AA, Clark AM (2011). Oxidative deoxyribonucleic acid damage in sperm has a negative impact on clinical pregnancy rate in intrauterine insemination but not intracytoplasmic sperm injection cycles. Fertil Steril.

[19] Anifandis G, Bounartzi T, Messini CI, Dafopoulos K, Markandona R, Sotiriou S (2015). Sperm DNA fragmentation measured by Halosperm does not impact on embryo quality and ongoing pregnancy rates in IVF/ICSI treatments. Andrologia.

[20] Bichara C, Berby B, Rives A, Jumeau F, Letailleur M, Setif V (2019). Sperm chromatin condensation defects, but neither DNA fragmentation nor aneuploidy, are an independent predictor of clinical pregnancy after intracytoplasmic sperm injection. J Assist Reprod Genetr.

[21] Humaidan P, Eleuteri P, Rescia M, Bungum M, Spano M, Spanò M (2008). Sperm chromatin structure assay parameters measured after density gradient centrifugation are not predictive for the outcome of ART. Hum Reprod.

[22] Barratt CLR, Aitken RJ, Björndahl L, Carrell DT, de Boer P, Kvist U (2010). Sperm DNA: organization, protection and vulnerability: from basic science to clinical applications–a position report. Hum Reprod.

[23] Schlegel PN, Sigman M, Collura B, de Jonge CJ, Eisenberg ML, Lamb DJ (2021). Diagnosis and treatment of infertility in men: AUA/ASRM guideline part I. Fertil Steril Fertil Steril.

[24] Practice Committee of the American Society for Reproductive Medicine (2013). The clinical utility of sperm DNA integrity testing: a guideline. Fertil Steril.

[25] Ribas-Maynou J, Yeste M, Becerra-Tomás N, Aston K, James E, Salas-Huetos A (2021). Clinical implications of sperm DNA damage in IVF and ICSI: updated systematic review and meta-analysis. Biol Rev Camb Philos Soc.

[26] Newman H, Catt S, Vining B, Vollenhoven B, Horta F (2022). DNA repair and response to sperm DNA damage in oocytes and embryos, and the potential consequences in ART: a systematic review. Mol Hum Reprod.

[27] Horta F, Catt S, Ramachandran P, Vollenhoven B, Temple-Smith P (2020). Female ageing affects the DNA repair capacity of oocytes in IVF using a controlled model of sperm DNA damage in mice. Hum Reprod Hum Reprod.

[28] Aly J, Plowden TC, Christy AY (2021). Factors contributing to persistent disparate outcomes of in vitro fertilization treatment. Curr Opin Obstet Gynecol NLM.

[29] Luo M, Li D, Xia M, Xie H, Liu P, Qin Y (2022). Blastocyst euploidy rates in low-prognosis patients according to the POSEIDON criteria: a retrospective analysis of 3016 embryos. Reprod Biomed Online.

[30] Blyth U, Craciunas L, Hudson G, Choudhary M (2021). Maternal germline factors associated with aneuploid pregnancy loss: a systematic review. Hum Reprod Update Hum Reprod Update.

[31] Huang Y, Tu M, Qian Y, Ma J, Chen L, Liu Y (2022). Age-dependent metabolomic profile of the follicular fluids from women undergoing assisted reproductive technology treatment. Front Endocrinol.

[32] Wyns C, de Geyter C, Calhaz-Jorge C, Kupka M, Motrenko T, Smeenk J (2018). ART in Europe, results generated from European registries by ESHRE. Hum Reprod Open.

[33] Tang X, Xiao Q, Wang X, He Y, Tian Y, Xia B (2022). Single-cell transcriptomics-based study of transcriptional regulatory features in the Non-Obstructive Azoospermia Testis. Front Genet.

[34] Cao N, Hu C, Xia B, He Y, Huang J, Yuan Z (2022). The Activated AMPK/mTORC2 Signaling Pathway Associated with Oxidative Stress in Seminal Plasma Contributes to Idiopathic Asthenozoospermia. Oxid Med Cell Longev.

[35] Yuan S, Schuster A, Tang C, Yu T, Ortogero N, Bao J (2016). Sperm-borne miRNAs and endo-siRNAs are important for fertilization and preimplantation embryonic development. Development.

[36] Cui L, Fang L, Shi B, Qiu S, Ye Y (2015). Spermatozoa micro ribonucleic acid-34c level is correlated with intracytoplasmic sperm injection outcomes. Fertil Steril Fertil Steril.

[37] Ribas-Maynou J, Garcia-Bonavila E, Bonet S, Catalán J, Salas-Huetos A, Yeste M (2021). The TUNEL assay underestimates the incidence of DNA damage in pig sperm due to chromatin condensation. Theriogenology.

[38] Mitchell LA, de Iuliis GN, Aitken RJ (2011). The TUNEL assay consistently underestimates DNA damage in human spermatozoa and is influenced by DNA compaction and cell vitality: development of an improved methodology. Int J Androl.

[39] Simon L, Aston KI, Emery BR, Hotaling J, Carrell DT (2017). Sperm DNA damage output parameters measured by the alkaline comet assay and their importance. Andrologia.

[40] Ribeiro SC, Muratori M, de Geyter M, de Geyter C (2017). TUNEL labeling with BrdUTP/anti-BrdUTP greatly underestimates the level of sperm DNA fragmentation in semen evaluation. PLoS One.

[41] Agarwal A, Barbăroșie C, Ambar R, Finelli R (2020). The impact of single- and double-strand DNA breaks in human spermatozoa on assisted reproduction. Int J Mol Sci.

[42] Casanovas A, Ribas-Maynou J, Lara-Cerrillo S, Jimenez-Macedo AR, Hortal O, Benet J (2019). Double-stranded sperm DNA damage is a cause of delay in embryo development and can impair implantation rates. Fertil Steril.

[43] Ribas-Maynou J, Benet J (2019). Single and double strand sperm DNA damage: different reproductive effects on male fertility. Genes.

[44] Lara-Cerrillo S, Ribas-Maynou J, Rosado-Iglesias C, Lacruz-Ruiz T, Benet J, García-Peiró A (2021). Sperm selection during ICSI treatments reduces single- but not double-strand DNA break values compared to the semen sample. J Assist Reprod Genet.

[45] Zhang Z, Dai C, Shan G, Chen X, Liu H, Abdalla K (2021). Quantitative selection of single human sperm with high DNA integrity for intracytoplasmic sperm injection. Fertil Steril Fertil Steril.

[46] Pastuszek E, Kiewisz J, Skowronska P, Liss J, Lukaszuk M, Bruszczynska A (2017). An investigation of the potential effect of sperm nuclear vacuoles in human spermatozoa on DNA fragmentation using a neutral and alkaline comet assay. Andrology.

[47] Torki-Boldaji B, Tavalaee M, Bahadorani M, Nasr-Esfahani MH (2017). Selection of physiological spermatozoa during intracytoplasmic sperm injection. Andrologia.

[48] Deng J, Zhao Q, Cinnioglu C, Kayali R, Lathi RB, Behr B (2020). The impact of culture conditions on blastocyst formation and aneuploidy rates: a comparison between single-step and sequential media in a large academic practice. J Assist Reprod Genet.

[49] Desai N, Yao M, Richards EG, Goldberg JM (2020). Randomized study of G-TL and global media for blastocyst culture in the EmbryoScope: morphokinetics, pregnancy, and live births after single-embryo transfer. Fertil Steril Fertil Steril.

[50] Ribas-Maynou J, Yeste M, Salas-Huetos A (2020). The relationship between sperm oxidative stress alterations and IVF/ICSI outcomes: a systematic review from nonhuman mammals. Biology..

[51] Cho C-L, Agarwal A (2018). Role of sperm DNA fragmentation in male factor infertility: a systematic review. Arab J Urol.

[52] Blázquez A, García D, Rodríguez A, Vassena R, Vernaeve V (2016). Use of donor sperm in addition to oocyte donation after repeated implantation failure in normozoospermic patients does not improve live birth rates. Hum Reprod Hum Reprod.

[53] Oehninger S, Chaturvedi S, Toner J, Morshedi M, Mayer J, Lanzendorf S (1998). Semen quality: is there a paternal effect on pregnancy outcome in in-vitro fertilization/intracytoplasmic sperm injection?. Hum Reprod Hum Reprod.

[54] French DB, Sabanegh ES, Goldfarb J, Desai N (2010). Does severe teratozoospermia affect blastocyst formation, live birth rate, and other clinical outcome parameters in ICSI cycles?. Fertil Steril Fertil Steril.

[55] Svalander P, Jakobsson AH, Forsberg AS, Bengtsson AC, Wikland M (1996). The outcome of intracytoplasmic sperm injection is unrelated to “strict criteria” sperm morphology. Hum Reprod Hum Reprod.

[56] Taşdemir I, Taşdemir M, Tavukçuoģlu Ş, Kahraman S, Biberoģlu K (1997). Effect of abnormal sperm head morphology on the outcome of intracytoplasmic sperm injection in humans. Hum Reprod Hum Reprod.

[57] Cissen M, van Wely M, Scholten I, Mansell S, de Bruin JP, Mol BW (2016). Measuring Sperm DNA fragmentation and clinical outcomes of medically assisted reproduction: a systematic review and meta-analysis. PLoS One.

[58] Simon L, Zini A, Dyachenko A, Ciampi A, Carrell D (2017). A systematic review and meta-analysis to determine the effect of sperm DNA damage on in vitro fertilization and intracytoplasmic sperm injection outcome. Asian J Androl Asian J Androl.

[59] West R, Coomarasamy A, Frew L, Hutton R, Kirkman-Brown J, Lawlor M (2022). Sperm selection with hyaluronic acid improved live birth outcomes among older couples and was connected to sperm DNA quality, potentially affecting all treatment outcomes. Hum Reprod Hum Reprod.

[60] Nuñez-Calonge R, Caballero P, López-Fernández C, Guijarro JA, Fernández JL, Johnston S (2012). An improved experimental model for understanding the impact of sperm DNA fragmentation on human pregnancy following ICSI. Reprod Sci.

[61] Simopoulou M, Sfakianoudis K, Maziotis E, Tsioulou P, Grigoriadis S, Rapani A (2021). PGT-A: who and when? α systematic review and network meta-analysis of RCTs. J Assist Reprod Genet.

[62] Bernstein KA, Rothstein R (2009). At loose ends: resecting a double-strand break. Cell.

[63] Simon L, Lutton D, McManus J, Lewis SEM (2011). Sperm DNA damage measured by the alkaline comet assay as an independent predictor of male infertility and in vitro fertilization success. Fertil Steril.

[64] Musson R, Gąsior Ł, Bisogno S, Ptak GE (2022). DNA damage in preimplantation embryos and gametes: specification, clinical relevance and repair strategies Hum Reprod Update.

[65] Gawecka JE, Marh J, Ortega M, Yamauchi Y, Ward MA, Ward WS (2013). Mouse zygotes respond to severe sperm DNA damage by delaying paternal DNA replication and embryonic development. PLoS ONE.

[66] Adiga SK, Toyoshima M, Shiraishi K, Shimura T, Takeda J, Taga M (2007). p21 provides stage specific DNA damage control to preimplantation embryos. Oncogene.

[67] Khokhlova Ev, Fesenko ZS, Sopova Jv, Leonova EI (2020). Features of DNA repair in the early stages of mammalian embryonic development. Genes.

[68] Aitken RJ, Gibb Z, Baker MA, Drevet J, Gharagozloo P (2016). Causes and consequences of oxidative stress in spermatozoa. Reprod Fertil Dev Reprod Fertil Dev.

[69] Olsen AK, Bjørtuft H, Wiger R, Holme J, Seeberg E, Bjørås M (2001). Highly efficient base excision repair (BER) in human and rat male germ cells. Nucleic Acids Res Nucleic Acids Res.

[70] Ribas-Maynou J, Gawecka JE, Benet J, Ward WS (2014). Double-stranded DNA breaks hidden in the neutral comet assay suggest a role of the sperm nuclear matrix in DNA integrity maintenance. Mol Hum Reprod.

[71] Turan V, Oktay K (2020). BRCA-related ATM-mediated DNA double-strand break repair and ovarian aging. Hum Reprod Update Hum Reprod Update.

[72] Stringer JM, Winship A, Liew SH, Hutt K (2018). The capacity of oocytes for DNA repair. Cell Mol Life Sci.

[73] Wdowiak A, Bakalczuk S, Bakalczuk G (2015). The effect of sperm DNA fragmentation on the dynamics of the embryonic development in intracytoplasmatic sperm injection. Reprod Biol.

[74] Esbert M, Pacheco A, Soares SR, Amorós D, Florensa M, Ballesteros A (2018). High sperm DNA fragmentation delays human embryo kinetics when oocytes from young and healthy donors are microinjected. Andrology.

[75] Wang S, Tan W, Huang Y, Mao X, Li Z, Zhang X (2022). Sperm DNA fragmentation measured by sperm chromatin dispersion impacts morphokinetic parameters, fertilization rate and blastocyst quality in ICSI treatments. Zygote.

[76] Baran V, Pisko J (2022). Cleavage of early mouse embryo with damaged DNA. Int J Mol Sci.

[77] Toyoshima M (2009). Analysis of p53 dependent damage response in sperm-irradiated mouse embryos. J Radiat Res J Radiat Res.

[78] Paul RC, Fitzgerald O, Lieberman D, Venetis C, Chambers GM (2020). Cumulative live birth rates for women returning to ART treatment for a second ART-conceived child. Hum Reprod Hum Reprod.

[79] Luke B, Brown MB, Stern JE, Missmer SA, Fujimoto VY, Leach R (2011). Female obesity adversely affects assisted reproductive technology (ART) pregnancy and live birth rates. Hum Reprod Hum Reprod.

[80] WHO. WHO Laboratory manual for the examination and processing of human semen 5th Editio World Health Organization, editor. World Health Organization Press. 2010, ISBN 9789241547789.

[81] Langie SAS, Azqueta A, Collins AR (2015). The comet assay: past, present, and future. Front Genet.

[82] Gardner DK, Lane M, Stevens J, Schlenker T, Schoolcraft WB (2000). Blastocyst score affects implantation and pregnancy outcome: towards a single blastocyst transfer. Fertil Steril Fertil Steril.

