# Supplementary material for: Sperm DNA integrity does play a crucial role for embryo development after ICSI, notably when good-quality oocytes from young donors are used
Source: Biol Res. 2022 Dec 26;55:41. doi: 10.1186/s40659-022-00409-y (PMC9791757; doi:10.1186/s40659-022-00409-y)
Supplement: Supplementary file 1 — Additional file 1: Table S1. Exclusion criteria for semen and oocyte donors. Table S2. Characteristics of sperm donors. [file 40659_2022_409_MOESM1_ESM.docx]

Additional file 1: Table S1. Exclusion criteria for semen and oocyte donors.

|  | **General exclusion** | **Psychological conditions** | **Reproductive medicine diagnosis** | **Laboratory tests** | **Temporary exclusions** |
| --- | --- | --- | --- | --- | --- |
| Oocyte donors | - Age < 18 or > 35 - BMI < 19 or > 28 - Myopia > 6 diopters - Having been adopted - > 6 descendants through ART or natural intercourse - Presence of hereditary diseases | - History of personal or familiar psychological disorders - Criminal records - Drug abuse - Deliberate and repeated inaccuracy in the information provided - Lack of consciousness regarding gamete donation | - Previous infertility history - Personal or familiar hereditary diseases - Uterine malformations - Low ovarian reserve (Antral follicle count < 10-15) | - Anti-müllerian hormone < 1.5 ng/mL - Positive for X-linked diseases - Altered karyotype - Serology for Hepatitis B or C, HIV, Human T-lymphotrophic virus positive - Cholesterol >300 mg/dL - Triglycerides > 250 mg/dL | - Presence of mammary nodules - Cytology: CIN2 or CIN3 - Positive for syphilis - Presence of teratomas, endometriomas and ovarian tumors - Hydrosalpinx - Taking medications that can affect gametes |
| Semen donors | - Age < 18 or > 35 - BMI < 18 or > 30 - Myopia > 6 diopters - Having been adopted - > 6 descendants through ART or natural intercourse - Presence of hereditary diseases | - History of personal or familiar psychological disorders - Drug abuse - Criminal records - Deliberate and repeated inaccuracy in the information provided - Lack of consciousness regarding gamete donation | - Presence of urogenital malformations - Previous infertility history | - Altered karyotype - Serology for Hepatitis B or C, HIV, Human T-lymphotrophic virus - Renal insufficiency - Diabetes - Triglycerides > 250 mg/dL - Cholesterol >300 mg/dL | - Positive for syphilis - Positive bacterial culture in urine or semen - Smoking >20 cigarette/day - Taking medications that can affect gametes |

Additional file 1: Table S2. Characteristics of sperm donors

|  | Value  (mean ± SD) |  |  |
| --- | --- | --- | --- |
| Height (m) | 1.80 ± 0.05 |  |  |
| Weight (kg) | 71.48 ± 7.78 |  |  |
| BMI | 22.14 ± 1.95 |  |  |
| Sport (hours / week) | 3.04 ± 1.34 |  |  |
| Smoking (cigarettes / day) | 1.89 ± 2.40 |  |  |
|  |  |  |  |
|  |  |  |  |
|  | NO | YES | Occasionally |
| Cigarette consumption | 55% | 35% | 10% |
| Coffee consumption | 32% | 68% | 0% |
| Alcohol consumption | 16% | 32% | 52% |
| Drug consumption | 81% | 0% | 19% |
